# Massive shelf dense water flow influences plankton community structure and particle transport over long distance

**DOI:** 10.1038/s41598-018-22569-2

**Published:** 2018-03-14

**Authors:** Fabrizio Bernardi Aubry, Francesco Marcello Falcieri, Jacopo Chiggiato, Alfredo Boldrin, Gian Marco Luna, Stefania Finotto, Elisa Camatti, Francesco Acri, Mauro Sclavo, Sandro Carniel, Lucia Bongiorni

**Affiliations:** 1 Institute of Marine Sciences, National Research Council, Arsenale - Tesa 104, Castello 2737/F, 30122 Venice, Italy; 2 Institute of Marine Sciences, National Research Council, Largo Fiera della Pesca 2, 60125 Ancona, Italy

## Abstract

Dense waters (DW) formation in shelf areas and their cascading off the shelf break play a major role in ventilating deep waters, thus potentially affecting ecosystem functioning and biogeochemical cycles. However, whether DW flow across shelves may affect the composition and structure of plankton communities down to the seafloor and the particles transport over long distances has not been fully investigated. Following the 2012 north Adriatic Sea cold outbreak, DW masses were intercepted at ca. 460 km south the area of origin and compared to resident ones in term of plankton biomass partitioning (pico to micro size) and phytoplankton species composition. Results indicated a relatively higher contribution of heterotrophs in DW than in deep resident water masses, probably as result of DW-mediated advection of fresh organic matter available to consumers. DWs showed unusual high abundances of *Skeletonema* sp., a diatom that bloomed in the north Adriatic during DW formation. The Lagrangian numerical model set up on this diatom confirmed that DW flow could be an important mechanism for plankton/particles export to deep waters. We conclude that the predicted climate-induced variability in DW formation events could have the potential to affect the ecosystem functioning of the deeper part of the Mediterranean basin, even at significant distance from generation sites.

## Introduction

The open-ocean dense water convection has long been acknowledged as substantial player in the global thermohaline circulation, while only recently the role of dense water formation over the shelf and cascading off the shelf break^1,2^ has been recognized. The dense shelf water cascading (DSWC) is a type of gravity current, occurring when dense water (DW) forms over the continental shelf due to atmospheric forcing (i.e. cooling or salification of coastal waters). Once formed those waters descend down the continental slope as near-bottom gravity current or as intermediate-depth intrusion^1^. Although more than 60 sites of cascading have been documented around the world, due to its highly intermittent nature both in time and in space, the wider-scale significance of this phenomenon has still to be quantified^1^. DSWC off the shelf break has been recognized as having a major role in bringing oxygen to deep water masses^3^ and, by providing cross-shelf transport of organic matter, nutrients, oxygen and organisms, it has the potential to change the biodiversity of deep-sea communities, affecting biogeochemical cycles, and influencing the overall deep-sea functioning^3–5^. Although some studies have documented episodic changes of abundance, biomass and composition of plankton along water column as result of winter convective events^6–10^, studies on the effects of shelf DW advection on the plankton communities are still insufficient. Yoder and Ishimaru^11^ reported unusual high concentrations of phytoplankton (especially of two diatoms, *Skeletonema costatum* and *Asterionella japonica*) in DW cascaded off the southeastern United States continental shelf, at depths higher than 100 meters. Recently Luna *et al*.^12^ demonstrated that the flow of dense currents over the sea floor increased the abundance of deep-sea prokaryotes, shaped microbial community composition and stimulated microbial biomass production and organic matter degradation rates. Filling this gap of knowledge, not just for few species but for the whole plankton community, is undoubtedly important, given that plankton organisms are the major agents of biogeochemical cycling and ecosystem functioning and account for a consistent fraction of marine biodiversity^13,14^. Moreover, planktonic organisms have shown to be extremely sensitive to climatic and oceanographic changes^15^. In light of the foreseen climate changes and its possible effects on the magnitude and frequency of DW formation^2^, it is of paramount importance to understand how those modifications will affect the ecosystem functioning and the energy flow across the trophic web, from surface to deep seas.

The Mediterranean Sea is a semi enclosed basin affected by strong interactions with the surrounding lands and for this reason is highly vulnerable to climate changes, providing an optimal case study for addressing the relationships between local climate, coastal oceanography and ecosystems effects^16^. Within the Mediterranean, the northern Adriatic Sea is one of the three generation sites of dense shelf water formation along with the Gulf of Lion and the northern Aegean Sea^17^. In this area, the severe heat loss and evaporation occurring during cold outbreaks associated to the Bora (a cold and dry north-easterly wind) densify the resident water mass producing the so-called Northern Adriatic Dense Water (NAdDW)^18–20^. This water mass leaves the area of generation as a buoyancy-driven, bottom-arrested current travelling along the western Adriatic shelf in nearly geostrophic equilibrium and, after 2–4 months, reaches the southern Adriatic where it sinks to the deep-sea intermittently through multiple cascading events^21–23^.

During February 2012, the European region experienced the most significant severe cold spell event of the past 30 years, which triggered significant heat loss in the northern Adriatic as well as in other northern Mediterranean shelves^24^. The cold air outbreak (CAO), associated to a severe cold and dry flow of northeasterlies^25^ and the very limited discharge of the Po River in the preceding fall, caused the formation of exceptional dense shelf water^2,22^. Following this event, an interdisciplinary, multiplatform, dedicated cruise (ODW 2012) was organized and water samples were collected in the southern Adriatic at about 460 km from the area of origin where the main cascading events take place. This rapid response cruise presented several logistical complexities due to a short planning time. Anyway, it gave the unique chance of investigating a massive stochastic event, that otherwise would have been only partially studied through moored or drifting instrumentations deployed before the event. The simultaneous collection of water samples with the DW passage was possible thanks to an ensemble of hydrodynamic models and Lagrangian simulations implemented right after the DW formation event. The positioning of each station was planned with the aid of those simulations before field activities, and operationally checked while off shore. To our knowledge, this innovative sampling approach, matching biology, oceanography and modelling, has been applied very rarely in biological studies. In our case, this was very effective for a precise selection of geographical positions and depths of sampling sites and the collection of distinct water masses, allowing us to explain complex interactions between physical and biological processes.

**Figure 1:**
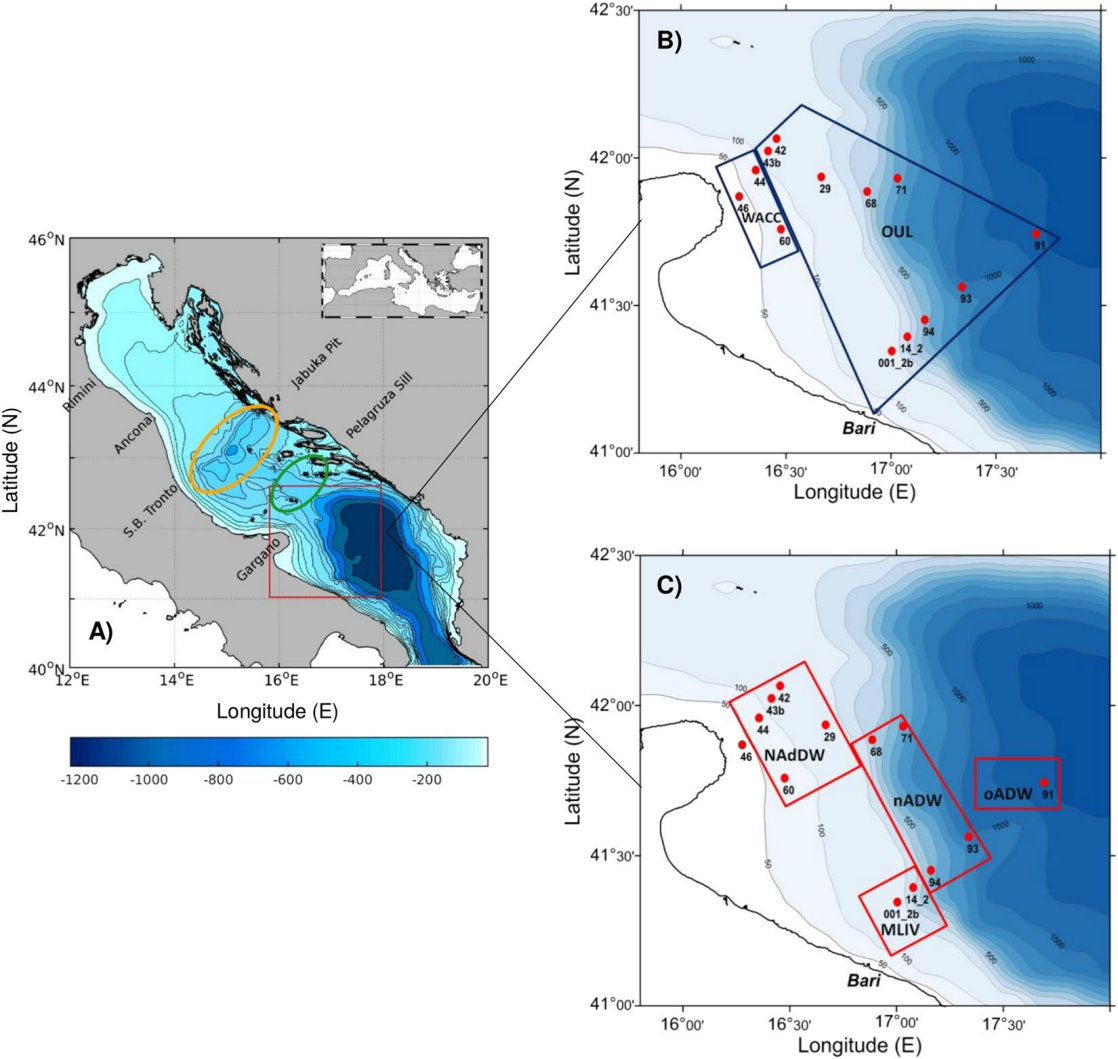
Map of the investigated area (red frame) in the southern Adriatic Sea (**A**). Maps showing the localization of the sampling stations at surface (**B**) and deep (**C**) water layers. The yellow and green circles in map A depict the areas occupied by the Jabuka Pit and the Pelagruza Sill, respectively. Water masses’ acronyms: Shelf (Shelf Water), WACC (Western Adriatic Coastal Current), OUL (Offshore Upper Layer), MLIW (Modified Levantine Intermediate Water), NAdDW (Northern Adriatic Dense Water), nADW (new Adriatic Deep Water) and oADW (old Adriatic Deep Water). For water masses definition see the text. Six additional samples collected at the subsurface Deep Chlorophyll Maximum at stations 42 and 43b (Shelf), 44 (WACC), and 91, 93 and 94 (OUL) are not showed in the maps. Maps generated by using MATLAB 8.5 http://uk.mathworks.com/products/matlab (map A) and ODV 4.7.10 (Schlitzer, R., Ocean Data View, http://odv.awi.de, 2017, maps B and C).

With the aim of investigating the influence of the DW flow on the structure of the plankton communities, from pico- to micro- size classes, 28 samples representing water masses of different origin (including DW and resident waters) were collected at 13 stations (Fig. 1). The partitioning of plankton biomass and the phytoplankton species composition of different water masses were compared to answer the following questions: i) does the flow of DW affect the biomass and relative contribution of the different plankton size classes in resident water masses? ii) are the phytoplankton diversity and community structure affected by DW advection? iii) could the dense water flow actively transport small particles from the NAdDW generation area to the cascading site? iv) are the high concentrations of *Skeletonema* sp. (up to 40% of phytoplankton abundance) found in dense water samples during the 2012 event connected with a phytoplankton bloom in the NA during the generation event?

## Results

### Definition of water masses in the study area

**Figure 2:**
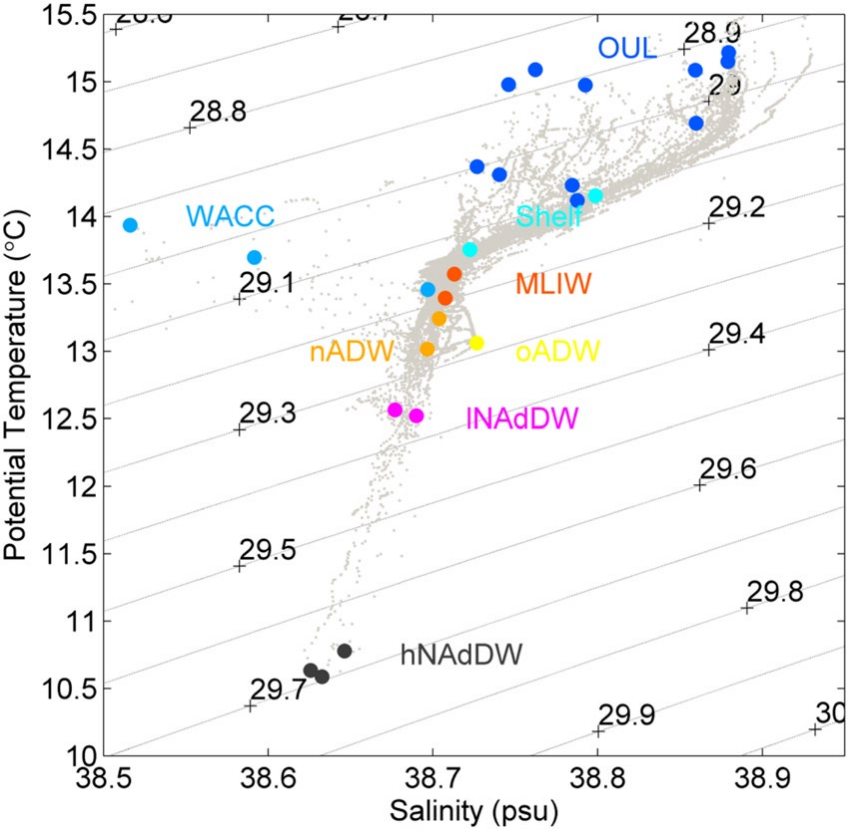
T-S plot of CTD casts; all casts (grey dots) on the background. On the foreground, coloured dots show the location on the T-S plot of the samples considered in this study. The associated coloured labels identify the water mass: Shelf (Shelf Water), WACC (Western Adriatic Coastal Current), OUL (Offshore Upper Layer), MLIW (Modified Levantine Intermediate Water), lNAdDW (Northern Adriatic Dense Water, low content), hNAdDW (high content), nADW (newly generated Adriatic Deep Water) and oADW (old, resident Adriatic Deep Water); numbers correspond to density anomaly (kg m^−3^); lines inside the plot are isopycnals of potential density anomaly (kg m^−3^).

Water mass characterization was performed based on temperature, salinity and dissolved oxygen following an equivalent classification used in Luna *et al*.^12^, with the inclusion of the Shelf Water to account for additional sampling depths considered here (Fig. 2, Supplementary Table S1). A full review of the water masses in the area is available in Chiggiato and colleagues^26^. Three distinct masses were identified in the surface and subsurface layers (Fig. 2, Supplementary Table S2): a low-salinity water of riverine influence (WACC, Western Adriatic Coastal Current, between 0.5 m and 15 m depth), a shelf water mass generally well mixed by wind and tides (Shelf; 15–26 m) and a higher-salinity, slightly warmer mass defined as Offshore Upper Layer (OUL, 0.5 m), most likely influenced by lateral advection of Ionian Surface Water and found at stations off the shelf. Near the sea floor, two types of NAdDW were intercepted: a slightly warmer and less dense (low content of NAdDW detected between 72 and 82 m depth, hereafter named lNAdDW) formed by NAdDW mixing with resident waters while travelling on the shelf and a relatively purer DW (high content of NAdDW, ranging between 113 and 141 m depth, hereafter named hNAdDW). Two stations intercepted the Modified Levantine Intermediate Water (MLIW) at depths between 133 and 173 m, a relatively old water mass with low dissolved oxygen content. The New Adriatic Deep Water (nADW) was found near the bottom at two stations (367 and 600 m). This water mass is relatively richer in dissolved oxygen than the resident one being generated by recent cascading of NAdDW turbulently mixed with MLIW along the descent. Old ADW (oADW), generated in previous years and therefore strongly depleted in dissolved oxygen, was found at the deepest station (1194 m).

### Plankton biomass partitioning

**Figure 3:**
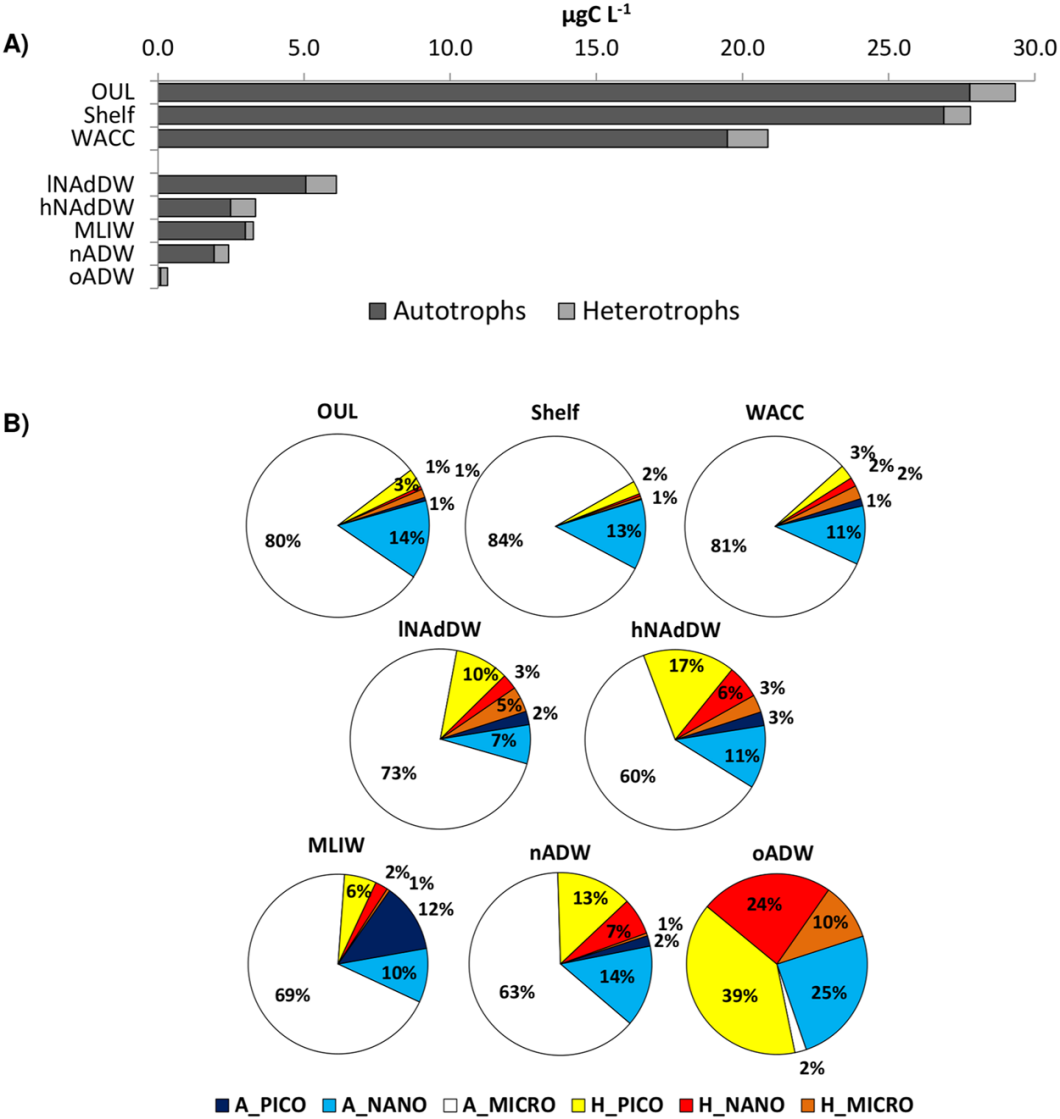
Autotrophic (dark gray bars) and heterotrophic (light gray bars) plankton biomass (**A**). Plankton biomass partitioning (expressed as percentage over the total biomass) in the different water masses (**B**); autotrophic pico-(A_PICO), nano-(A_NANO), micro-(A_MICRO), heterotrophic pico-(H_PICO), nano-(H_NANO), micro-(H_MICRO) plankton.

Total plankton biomass (from pico to micro size) showed a decreasing trend from the surface (on average 29.3 μgC L^−1^) to the deepest water masses (0.3 μgC L^−1^; Fig. 3A, Supplementary Table S2). The plankton biomass partitioning (contribution of autotrophic vs heterotropic pico, nano and micro organisms size-classes, details in Materials and Methods), showed a clear difference between surface and deep waters (Fig. 3B), as confirmed by ANOSIM (Global R = 0.921, *p* < 0.001). All surface and subsurface water masses were characterized by the dominance of phytoplankton biomass (average 95%, mainly large diatoms in the range between nano and micro-size) and by low biomass of heterotrophs (average 5%, mainly picoplankton, Fig. 3B). Deep waters displayed a comparatively higher contribution of heterotrophs (range 9–73%, Fig. 3B); this was reflected in a higher heterotrophs to autotrophs ratio (H/A) compared to surface/subsurface waters (Supplementary Table S2). The deepest oADW showed peculiar characteristics: lowest plankton biomass and autotrophic contribution (27% of biomass, mainly represented by nanoflagellates and benthic resuspended nano-sized diatoms), and the prevalence of heterotrophs (73%). In this sample, heterotrophs were dominated by small pico- and nanoplankton (39% and 24% of whole biomass respectively); moreover, microzooplankton contribution (10%, mainly eggs, copepods nauplii and non-loricate ciliates) was the highest of the whole dataset. When excluding oADW, the other deep water masses (including DWs) shared similar plankton composition and maintained distinctive characteristics in comparison to shallow waters: a relatively lower average biomass contribution of autotrophic microplankton (67% as opposed to 82% in surface/subsurface waters) and a higher percentage of heterotrophic pico- (average 11%), nano- (average 4%, mainly nanoflagellates and dinoflagellates of the genus *Gyrodinium* spp.) and microplankton (average 2%, Fig. 3B, Supplementary Table S2). It is worth mentioning that among deep waters, both NAdDW and nADW displayed a higher contribution of heterotrophs (higher H/A) than the MLIW which in turn hosted the highest contribution of pico-autotrophs (12%). Moreover microzooplankton in the NAdDW reached up to 5% of the total biomass (7% at station 29 where the densest waters were intercepted), while this component was almost absent in nADW and MLIW.

### Phytoplankton species composition

The phytoplankton community in the analysed water masses was composed by a total of 178 taxa: diatoms (97 taxa), dinoflagellates (51), coccolithophorids (18), Chrysophyceae (3), Euglenophyceae (3), Prasinophyceae (2), Cryptophyceae (2), silicoflagellates (1) and nanoflagellates (1).

**Figure 4:**
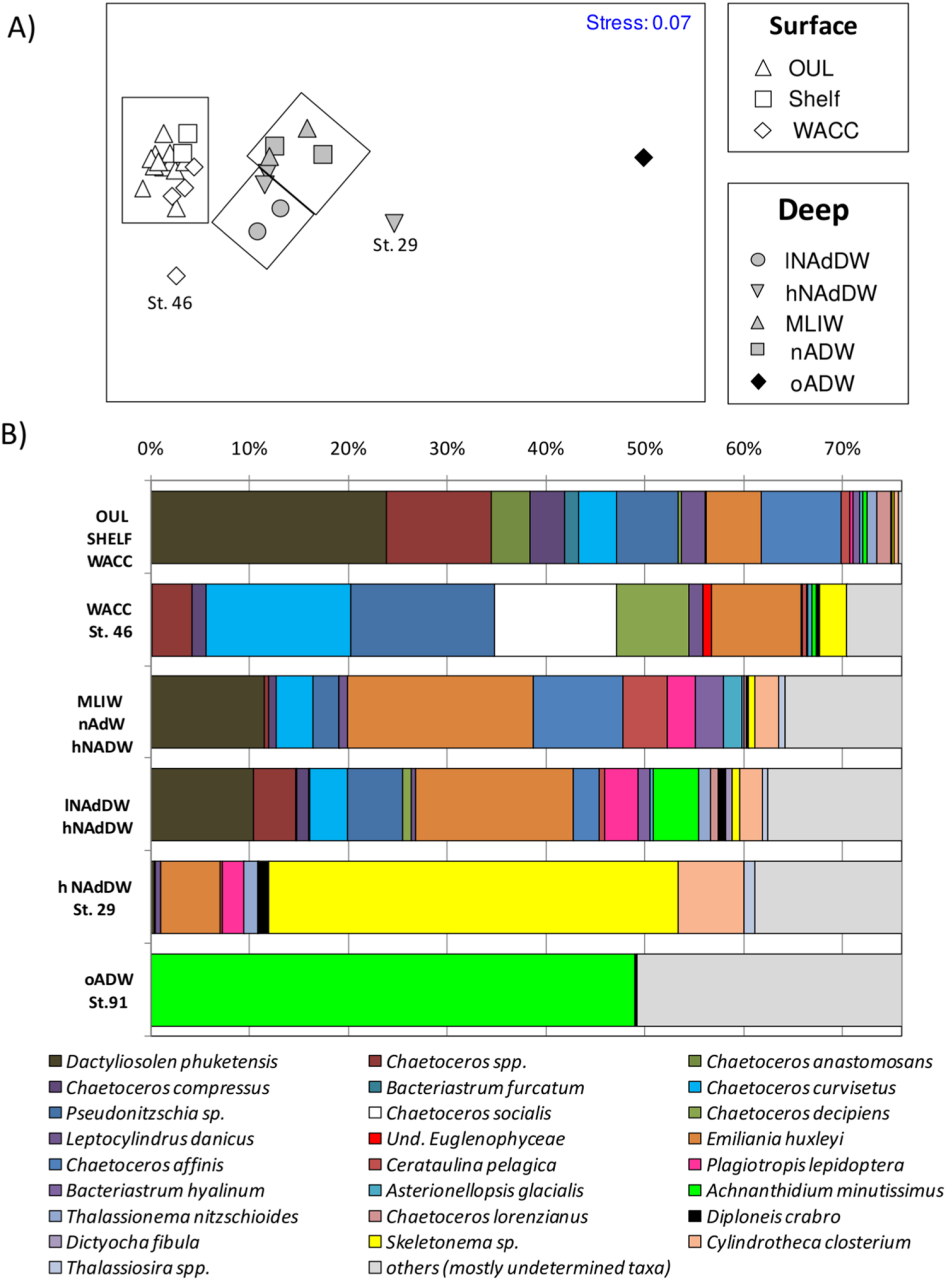
Non metric Multidimensional scaling (NMDS) of phytoplankton species composition in different water masses (**A**), each point symbolizes a sample and different symbols correspond to different water masses. Percentage composition of the most representative phytoplankton species (total species abundance accounting for >50%) characterizing each water mass and samples highlighted by NMDS (**B**). Data are reported as relative abundance; undetermined taxa (Cryptophyceae, coccolitophorids, nanoflagellates, dinoflagellates) are reported as others.

NMDS ordination showed a significant separation of samples according to water masses (ANOSIM test, Global R = 0.760, *p* < 0.001; Fig. 4A). The deepest oADW, characterized by the lowest number of species and phytoplankton abundance (mainly represented by the benthic diatom *Achnanthidium minutissimus*), showed the highest dissimilarity with the other water masses (between 68% and 95%). Among surface/subsurface waters the coastal station 46 (WACC) showed a clear separation from the other surface/subsurface samples (63% of dissimilarity). A clear separation occurred also between all surface/subsurface water masses and the deep water masses (69–82%). Among deep waters, lNAdDW grouped apart from MLIW, nADW and hNAdDW (50% of dissimilarity), while station 29 corresponding to hNAdDW (where the coldest and densest water mass of the whole study was intercepted)^26^ was very dissimilar respect to the other masses (62–91%, Fig. 4A).

Differences between water masses or sampling stations were mainly driven by marked dissimilarities in the relative abundance of some of the dominant taxa (such as *Dactyliosolen phuketensis*), and by the presence of taxa that were associated, albeit to different degree, only with certain water masses (Fig. 4B). Phytoplankton communities of surface waters were characterized by the presence of distinctive small colonial diatoms belonging to the nano-fraction (mainly the colonial diatoms *Chaetoceros* spp.). Most of these species were often associated with relatively large (up to 50 µm) colonial species (*Pseudo-nitzschia* sp.). Surface waters (WACC, especially st. 46) contained, in addition, species such as *Chaetoceros socialis* or Euglenophyceans which are typically found in waters influenced by coastal inputs. Conversely, deep-water masses were characterized by the presence of the benthic diatom *Diploneis crabro* and by higher contributions of the benthic and the epipelic diatoms *Plagiotropis lepidoptera* and *Cylindrotheca closterium* (up to 3% and 7% of total abundance, respectively). Differences among deep-water masses were mainly determined by different percentage of the pelagic diatoms *Chaetoceros affinis* and *Cerataulina pelagica* (more abundant in MLIW and nADW) or by benthic species as *A*. *minutissimus*, more abundant in lNAdDW, hNAdDW and oADW (Fig. 4B). The densest water sample (st. 29) showed a distinct composition, characterized by the higher contribution of the benthic diatoms *C*. *closterium* and *D*. *crabro* (7 and 1.1%, respectively) and, more significantly, by *Skeletonema* sp. contributing in this sample for up to 40% of the total abundance (Fig. 4B).

### Lagrangian particles transport and dense water export

**Figure 5:**
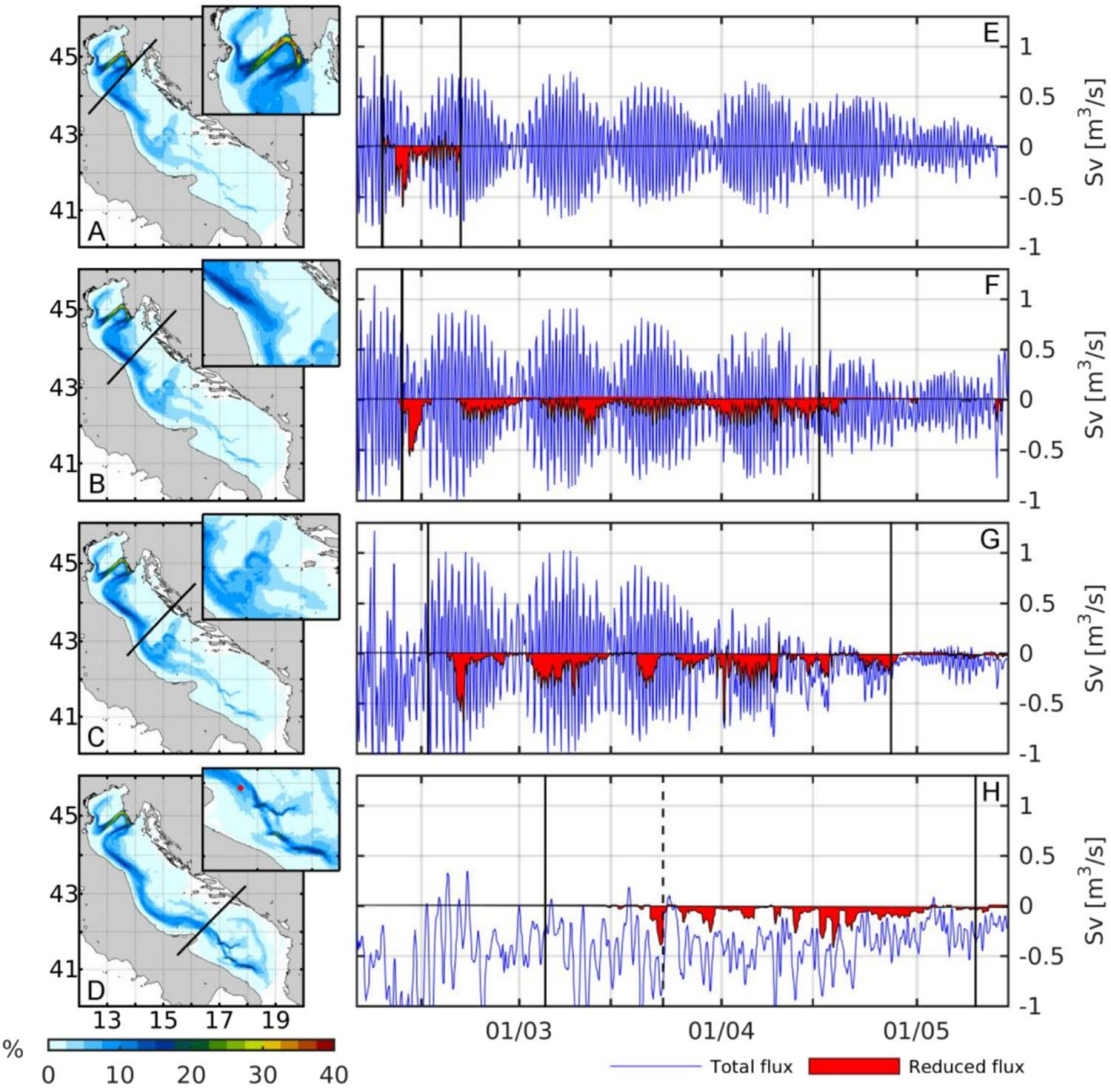
VDs pathways distributions (left panels) and fluxes (right panels) computed over particles passing across the four transects: Rimini (TR, panels A/E), Ancona (TA; panels B/F), San Benedetto del Tronto (TB, panels C/G) and Gargano (TG, panels D/H). Transects location are shown by black lines on the corresponding distribution map. Colour scale shows the percentage of passages computed over each subset. Red dot in the insert of panel D shows the location of station 29; blue lines show the total dense water (i.e. density ≥1029.3 kg m^−3^); red patches are the flux of the VDs carrying dense waters; positive/negative values represent northward/southward water fluxes. Vertical black lines mark the passages of the first VDs and of the cumulative 95% percentile of passages across each transect; Dashed line on panel H marks the sampling date for station 29 (figure generated by using MATLAB 8.5 http://uk.mathworks.com/products/matlab).

A Lagrangian model was implemented with virtual drifters having the buoyancy characteristics of *Skeletonema* sp., a diatom typically blooming in the north Adriatic during winter, which was found to be particularly abundant in high dense waters samples during the 2012 event. To investigate the patterns of distribution and transport of *Skeletonema* sp. during the 2012 CAO event, 200.000 Lagrangian virtual drifters were released in the northern Adriatic on the 5^th^ of February, coincident with a phytoplankton bloom occurred in this area. The drifters had buoyancy characteristics and dimensions similar to *Skeletonema* sp. (details in Materials and Methods) and hereafter will be referred to as Virtual Diatoms (VDs). The distribution of drifters concentrations computed for particles passing through 4 reference-transects (Fig. 5A–D) showed that, from the source area in the northern Adriatic Sea, VDs mostly follow pathways common to those of DW (see e.g. ref.^20^). In order to follow VDs leaving the source area during or right after *Skeletonema* sp. bloom, only drifters crossing a transect located in front of Rimini (TR, Fig. 5A) in less than 15 days from their release were considered. The VDs passing TR followed a major route that crossed the basin eastward toward the Istrian peninsula and then back in front of Rimini. Pathways of VDs through Ancona and San Benedetto del Tronto transects (TA and TB, Fig. 5B,C, respectively) showed two interesting features: a westward offshore expansion just south of TA related to the presence of a local gyre (not shown), and a high concentration of tracks falling into the Jabuka Pit and partially getting out through the Palagruža Sill (Fig. 1A). The VDs that crossed the southernmost transect (TG, Fig. 5D) located over the Gargano promontory and station 29, reached the southern Adriatic without falling into the Jabuka Pit and passed closer to the Italian coast. The simulation revealed that after passing TG, with highest pathways concentration pointing right over station 29, VDs either fell into the southern Adriatic Pit (along the Dauno Seamount, through the Bari Canyon system or off the Apulian shelf break) or exited the basin moving across the Apulian shelf.

The total flux (FT) of dense water (≥1029.3 kg m^−3^) computed every 6 hours across whole transect area, was clearly influenced in all transects by tidal modulation, shifting back and forth between positive (i.e. northward) and negative (southward) fluxes (Fig. 5E–H, blue line). An exception to this was TG where the modulation was still present but less clear. The flux of dense water specifically carrying VDs (Reduced Flux, FR) was less influenced by tides and in all transects showed an almost constant negative flux, with just some small exceptions (Fig. 5E–H, red patch). Number of particles and DW volume were computed for time spanning between zero and the 95^th^ percentile of the cumulated VDs passages for each transect (Table 1). In the three northernmost transects (TR, TA and TB) the FR volume was always significantly higher than the FT, while at TG this condition was inverted, and the volume of FT was over an order of magnitude higher than FR.

**Table 1:** Number of VD particles and total dense water volume crossing each transect (computed for the intervals shown in Fig. 5: in the case of TR a window of 15 days after drifters release was used, while for the other transects the first and 95^th^ percentile of the cumulative drifters’ passage was used). In parenthesis are reported the percentages over the total released VDs. Transects: Rimini (TR), Ancona (TA), San Benedetto del Tronto (TB) and Gargano (TG). Total and Reduced Flux (TF and RF).

	**Total VDs (% of released)**	**VDs in dense water (% of released)**	**TF dense water volume [km** ^3^ **]**	**RF dense water volume [km** ^3^ **]**
TR	12236 (6.18%)	12222 (6.11%)	0.4	−89.9
TA	9881 (4.94%)	9734 (4.87%)	−155.4	−536.7
TB	8437 (4.21%)	8203 (4.10%)	−500.8	−639.4
TG	3930 (1.96%)	3487 (1.74%)	−2283.6	−356.6

The phytoplankton bloom that developed in February 2012 during DW formation in the northern Adriatic presented high concentrations of *Skeletonema* sp. (up to 5 × 10^6^ cells L^−1^, Bernardi Aubry unpubl. data). Blooms of this species are common features of the area during winter season^27–30^. Considering 5 × 10^6^ cells L^−1^ as starting concentration, the simulated DW export of *Skeletonema* sp. computed between the passage of the first VD and the 95^th^ percentile (respectively on February 9^th^ and 21^st^) through TR amounted to 4.6 × 10^20^ cells. Given that in the numerical simulations 28.53% of VDs crossing TR arrived at TG, we could estimate a total number of 1.3 × 10^20^ VDs crossing TG. In particular the number of simulated VDs crossing station 29 (340), in an interval of ±2 days from the date this station was sampled, corresponded to 2.78% of the VDs leaving TR and to a total of 1.25 × 10^19^ cells (average concentration, 2.8 × 10^5^ cell L^−1^).

## Discussion

It is generally accepted that physical processes, such as mesoscale circulation, can influence the plankton community structure, by supply limiting nutrients to the euphotic zone in oligotrophic waters and stimulating planktonic responses such as productivity and changes in community structure^31,32^. Episodic events such as dense shelf water formation and cascading to the deep-sea have been recognized to significantly contribute to the ventilation of deep waters and to the overall ocean circulation^1^. Moreover, DSWC can fuel the deep water with large amounts of suspended particles, and organic matter, therefore potentially influencing the whole water column ecosystem functioning^5,33,34^. Despite this, few studies have considered the effects of DW flow on plankton community structure.

In this study NAdDW volumes formed as consequences of the 2012-CAO event were compared to resident waters in term of plankton biomass partitioning (pico to micro size) and phytoplankton species composition. Results showed that in all the water masses analysed (with the exception of the deepest oADW in which heterotrophy clearly prevailed), the plankton communities were dominated by autotrophic organisms (range between 54% and 98% of the total biomass). Although the massive presence of autotrophs in deep layers have been previously documented, and linked to the passive downslope-transport of surface cells facilitated by NAdDW^35^, in our study such characteristics was not exclusive of DWs but was general even for resident deep water masses. In February 2012 an intense winter convective episode, influencing the whole water column through vertical mixing processes, was registered in the South Adriatic Pit and in its surroundings^10^. Such hydrographic process, known to be responsible for the transport of nutrients from deep-sea reservoirs to the surface and of organic matter from the surface to deep waters, could explain the occurrence of high biomass of autotrophic organisms even in the aphotic layers, up to 400–600 m depth^7^. The ratio between planktonic heterotrophs and autotrophs (H/A) was higher in deep compared to surface/subsurface waters and was directly correlated with inorganic nutrients concentrations (Si-SiO_4_, R^2^ = 0.82; P-PO_4_, R^2^ = 0.62, N-NO_3_, R^2^ = 0.56, p < 0.001). While in the surface water masses, the presence of low nutrients can be the direct consequence of intense nutrients uptake by phytoplankton, in deep waters, the relatively higher nutrients concentrations coupled with higher H/A ratios could suggest the prevalence of remineralization processes mediated mainly by the heterotrophic picoplankton community. Interestingly, the comparison among deep water masses (after the exclusion of the oADW) highlighted that both NAdDW (lNAdDW and hNAdDW) and nADW were characterized by higher H/A ratios compared to the resident MLIW, mainly as results of the higher contribution of the smaller pico- and nano-heterotrophs. It is worth noticing that NAdDW and nADW reflect respectively conditions of pre-cascading and post-cascading or transition to post-cascading, being nADW here defined as younger water mass generated by recent cascading of NAdDW and turbulently mixed with MLIW along the descent. In agreement with those findings, Luna and colleagues^12^ detected high microbial abundances and enhanced secondary productivity in NAdDW compared to other resident water masses and ascribed these results to the availability of organic and inorganic nutrients. Mechanisms of enhanced marine organic carbon export from the continental shelf during cascading events have been extensively documented in the Adriatic and in the Gulf of Lions^3,34,36,37^. During 2012 the NAdDW reaching the Southern Adriatic were clearly characterized by higher concentrations of suspended matter and turbidity, higher organic and inorganic nutrients and oxygen concentrations compared to other deep resident waters^12^ (Supplementary Table S2). Moreover, data collected from sediment traps deployed in the cascading area during the 2012 event showed that organic particles in transit in the water column during DW flux-peaks, were mainly composed (~60%) by marine phytodetritus^23^. These data coupled with high chlorophyll *a* concentrations^23^ suggest that a non negligible amount of fresh and more bioavailable organic material was made available to planktonic consumers. We hypothesize here that the transport of fresh organic materials together with the increase in prokaryote biomass in DW veins^12^ could be responsible for triggering grazing activities. As support of this, we found a detectable increased contribution of nano- and micro-sized bacterivores/grazers (mainly heterotrophic flagellates and microzooplankton, Fig. 3B) to the total plankton biomass in both dense and nADW. Moreover, an increasing contribution of herbivores (mainly aloricated ciliates, titinnids, nauplii of copepods) was observed in the hNAdDW.

Regarding the phytoplankton communities, we found that NAdDW were characterized by a mix of benthic, epilitic and planktonic species. We hypothesize that this condition could reflect the combination of several passive physical processes: i) the mediated DW transport of surface species from the northern to the southern Adriatic Sea, as shown by numerical results; ii) the resuspension of bottom species due to bottom frictions during DW descent on the slope; iii) the mixing of dense and resident water masses along the path and during cascading (e.g. MLIW, nADW). The presence of benthic diatoms (e.g. *Plagiotropis lepidoptera* and *Diploneis crabro*), common in most deep water samples but more abundant in DW or exclusive of DW (such as the rare species, *Diploneis bombus*, <1%, not shown) could be taken as an indicator of turbulent mixing or DW energetic flow along the shelf and its friction with the bottom of the shelf break. Moreover, the presence of common species in dense and surface water masses (e.g., the diatom *Dactyliosolen phuketensis* occurring in OUL and WACC) could reflect the mixing of DW with adjacent surface water masses along their path. Besides this, the most evident consequence of the DW flow on the phytoplankton species composition was the finding of an unusual high contribution of the diatom *Skeletonema* sp. (up to 40% of total phytoplankton abundance) at station 29, where the coldest and densest water mass of the whole study was intercepted. This species is well known to bloom regularly in nutrient-rich, surface waters of the northern Adriatic Sea during February-March^27–30,38,39^, in coincidence with the late winter-spring start of its growing phase. The high contribution of *Skeletonema* sp. in the deep waters of the south Adriatic Sea, well below the euphotic layer, might thus be related to the export operated by the DW from the northern Adriatic source area. This hypothesis is highly consistent with the occurrence, during February 2012, of a bloom of *Skeletonema* sp. in the northern Adriatic and in the Venice Lagoon (Bernardi-Aubry pers. comm.), and the observation of a widespread bloom of chlorophyll *a* in the surface waters (satellite image data and biogeochemical modelling results, Supplementary Fig. S3). While before the 2012-CAO event a large fraction of the chlorophyll *a* was confined in the coastal waters, the onset of severe northeasterlies (typically setting up a double-gyre circulation in the area)^40–42^ was most likely able to spread the chlorophyll *a*, and arguably *Skeletonema* sp., all over the northern Adriatic Sea, probably covering most of the DW formation area. *Skeletonema* cells found in hNADW appeared morphologically intact, leading to hypothesize their origin from sinking of a local surface bloom. However, this option can be excluded since: i) we could not find any evidence of a local phytoplankton bloom in the area and in the days right before the sampling; ii) a high diatoms abundance was detected exclusively at station 29, where the densest waters were intercepted, whereas a low abundance or even no trace of the presence of *Skeletonema* was found in shallow or other deep-sea water masses (this conclusion is further supported by the finding, only at this station, of 16S rDNA sequences associated to diatoms plastids)^12^; iii) the density gradient at interface between the dense water vein and the ambient water reduces the mixing between the two water masses, and hence high concentrations of diatoms could have not reached the core of the vein that was sampled at station 29. Results of the hydrodynamic model and the Lagrangian simulation performed using VDs showed that, right after DW formation, large amounts of *Skeletonema* sp. cells left the north Adriatic Sea and were transported along the DW vein for about 460 km to the southern Adriatic, passing in front of the Gargano peninsula with high concentration near station 29 (Fig. 5). The model correctly places few VDs outside the dense water vein (Supplementary Fig. S5) in accordance with low values of diatoms found in non-DW stations.

The analysis of phytoplankton collected by sediment traps right downstream one of the main cascading hot-spot in the southern Adriatic revealed *Skeletonema* trapped at 700 m depth (Bernardi Aubry unpubl. data) together with a high amount of non living particles and a high contribution of organic carbon of marine origin^23^, clearly linking DW particle transport with the export of *Skeletonema* to the southern Adriatic Sea. Using a numerical Lagrangian model we estimated that within the two weeks following the bloom, about 28% of the cells leaving the northern Adriatic were exported to south by NAdDW. This percentage is lower than the one calculated only for water (up to 50% of the whole northern basin water mass was renewed during the whole CAO event)^22^, and this can be explained by the fact that only the densest waters are actually transporting particles that will reach the southern Adriatic Sea. This feature has also a direct effect on the amount of exported particles and on their traveling speed. In fact, particles carried by the denser waters travel with the fastest part of the vein (up to 1 m s^−1^) and hence are less affected by tidal modulation. This results in a more constant delivery of particles, which rarely can reverse their direction flux (instead common for the DW vein). Modelling showed that about 1.74% of VDs, corresponding to 2.8 × 10^5^cell L^−1^, could potentially reach the TG. Instead, samples collected at station 29 showed a concentration of 2.7 × 10^4^ cells L^−1^. The one order-magnitude overestimation produced by the Lagrangian model can be explained by the fact that VDs are conservative compared to natural cells, i.e. not subjected to grazing, degradation or other diluting ecological processes. Nevertheless, our simulation is still significant because it proves that during the 2012 event the NAdDW acted as a direct mean of transportation of diatoms from the northern to the southern Adriatic. In our numerical experiments *Skeletonema* sp. was used as model particle as this diatom was effectively observed at station 29, but on a broader point of view other particles with similar dimensional/buoyancy characteristics, e.g. microplastics^43^ or even dissolved substances (i.e. nutrients, heavy metals, pollutants) can be potentially transported by DWs.

Our results highlight how the NAdDW plays a fundamental role in connecting the northern and southern Adriatic subregions, in terms of not only water or dissolved oxygen, but also as far as dissolved substances and small particles are concerned. This is significant also in the perspective of ongoing climate dynamics; indeed, DW formation events are strongly connected to forcing (i.e. CAO-like events) and preconditioning conditions (i.e. temperature and salinity characteristics of the basin prior to the formation period), and both these aspects are greatly influenced by climate changes. In this regards, not only it is generally accepted that the Adriatic Sea will present saltier and warmer waters, as a consequence of higher atmospheric temperatures and decreases in riverine discharges^2,44^, but it is also expected that the number of CAO events will decrease^45^ and change in intensity. This will result in variation of the NAdDW not only in frequency and in magnitude of production, but also in its composition (i.e. its temperature and salinity) and on its ability to carry dissolved and small particulate matter.

Here by providing a snapshot of plankton communities composition in DW masses collected along their descent, at different levels of mixing with resident waters and within these latter, we demonstrated that DW flow can affect plankton species composition and biomass partitioning. We hypothesized that this can be exerted either through DW transport of allochthonous organic material, nutrients and oxygen, responsible for triggering heterotrophic processes in deep water layers, or through the export, even for long distance, of planktonic cells. Composition and size-distribution of plankton assemblages are major biological factors affecting the functioning of pelagic food-webs and consequently the rate of carbon export from the upper ocean to deep layers^46,47^. Results from this study highlight the potential of dense shelf water flow to influence the distribution and composition of the winter deep plankton communities. We furthermore underline the usefulness of combining plankton analysis and numerical modelling to trace oceanographic and ecological processes interaction.

## Materials and Methods

### Study area and sampling activities

Right after the onset of the CAO (23 March − 2 April 2012), a rapid response field campaign was carried out on board the R/V Minerva Uno. Thanks to the implementation of a pre-cruise numerical modelling ensemble, an adaptive planning was used to define sampling stations and DW masses were successfully intercepted in the southern Adriatic over 460 km from the area of origin (Fig. 1A; see ref.^26^ for more details). To characterize all water masses, samples were collected from 13 stations (in 9 stations at both surface and bottom depths and in 4 stations at surface or bottom, Fig. 1). Additional samples (6 stations) were collected at the subsurface layer corresponding to the deep chlorophyll maximum (DCM). Overall, 28 water samples were collected for the entire study (Supplementary Table S1). At each station and water depth, temperature, salinity, dissolved oxygen and turbidity were measured and seawater samples were collected with a CTD-rosette system consisting of a CTD SBE 911, coupled with a with SBE-43 sensor (resolution 4.3 μM) for dissolved oxygen measures, a Seapoint Turbidity Meter, and a General Oceanics rosette with 12 Niskin Bottles (having the capacity of 12 L each). Seawater samples for chlorophyll *a*, dissolved inorganic nutrients and total suspended matter (TSM) were immediately filtered through Whatman GF/F fibreglass filters (nominal porosity = 0.7 µm) and stored frozen. Samples for pico- and nanoplankton (0.2–2 and 2–20 µm cell size, respectively) counts were fixed with pre-filtered (0.2 µm) buffered formalin and glutaraldehyde, respectively, at 2% final concentration for both fixatives, and kept in the dark at 4 °C until processing. For microphytoplankton (>20 µm), seawater samples were fixed in acid Lugol’s iodine (1%) until further analyses. Seawater samples for microzooplankton (>20 µm) counts were preserved with neutralized formaldehyde (2.5% final concentration).

### Chlorophyll a, nutrients and TSM concentrations

Samples for chlorophyll *a* were analysed according to Holm-Hansen *et al*.^48^. Dissolved inorganic nitrogen (DIN: sum of ammonia, nitrite and nitrate), orthophosphates and orthosilicates were analysed with a Flow- Solution III autoanalyser (OI-Analytical), according to Strickland and Parsons^49^ and Hansen and Koroleff^50^. TSM was analysed according to Strickland and Parsons^49^.

### Abundance and biomass of auto- and heterotrophic picoplankton

Seawater sub-samples (4–30 ml) were filtered onto 0.2 µm pore size black polycarbonate filters. Total picoplankton counts followed the procedure by Noble and Fuhrman^51^. Filters were stained with SYBR Green (1:20 stock solution) in the dark for 15 min, rinsed and mounted on microscope glasses with a drop of 50% phosphate buffer (pH 7.8), glycerol and ascorbic acid (0,5%). Epifluorescent microscopy (EFM, 1000×) counts were performed by observing at least 20 microscopic fields and a minimum of 400 cells. For autotrophic picoplankton counts a set of unstained filters were directly observed using the EFM method. Heterotrophic picoplankton cells numbers were obtained by differences between total and autotrophic picoplankton counts. Three replicates were analysed for each sample. Auto- and heterotrophic picoplankton abundances were then converted into biomass using 250 fg C cell^−1^ and 20 fg C cell^−1^, respectively^52,53^.

### Abundance and biomass of auto- and heterotrophic nanoplankton

For auto- and hetrotrophic nanoplankton counts, water samples (50 mL) were filtered onto 0.8 µm pore size Nucleopore black membranes, stained with fluorescein isothiocyanate (FITC)^54^ and accurately rinsed before being mounted on microscope slides for EFM analyses. Counts were performed, at 1000× by observing longitudinal and latitudinal transects of filters, for a minimum of 200 cells. For autotrophic nanoplankton counts a set of unstained filters were directly observed using the EFM method, assuming that those displaying autofluorescence were autotrophic or mixotrophic cells. Heterotrophic nanoplankton cells numbers were obtained by differences between total and autotrophic nanoplankton counts. Three replicates were analysed for each sample. Whenever possible cells shape, presence of cilia and flagella and position of *sulcus* and *cyngulum* allowed the separation into the following taxonomic groups: flagellates, ciliates, coccolithophorids, naked or armoured dinoflagellates, Prasinophyceans, Cryptophyceans-, *Gymnodinium*-, *Gyrodinium*-, small *Navicula*-, *Nitzschia*-, *Skeletonema*-, and *Chaetoceros*-like cells. The maximal length and width of all nanoplanktonic cells were measured with a micrometric eyepiece or using an image analysis and the individual biovolume was estimated by approximating cell shape to geometric models^55^. Biovolumes were converted into biomass values using 220 fg C μm^−3^ as conversion factor for flagellates^56^, and for the others by the equations reported in Menden-Deuer and Lessard^57^.

### Abundance, biomass and species composition of phytoplankton

Phytoplankton was recognized and counted with an invertoscope (Axiovert 35, Zeiss) equipped with phase contrast^58,59^. A representative number of transects crossing the whole surface of the sedimentation chamber were screened at 400× and up to the whole chamber area was observed at 200×; one replicate was considered for each sample. Species composition was defined in accordance with Tomas^60^, Bérard-Therriault *et al*.^61^ and reference therein. Cell size was used for division between the nano (<20 µm) and the micro fractions (>20 µm). Division between microautotrophs and microheterotrophs was made based on known functional characteristics of taxa. Autotrophic dinoflagellates were counted after excluding typical heterotrophic taxa like *Protoperidinium*, *Gyrodinium*, *Diplopsalis* group, *Lessardia elongata* etc. Cells biovolumes were measured by approximating species shapes to geometrical models, and cell carbon content was calculated from mean cell-biovolume according to Stratmann^62^. Biomass estimates were computed using literature based C conversion factors. This likely leads to an over-estimation since during the 3 to 4 weeks DW transport diatoms would be presumably in degrading condition, and hence their C content should be decreasing. At the same time a reduced conversion factor cannot be proposed because the diatoms’ C content and stage of degradation cannot be properly assessed.

### Abundance, biomass and structure of microzooplankton

Seawater samples (5 L) were settled for 72 h, and concentrated to 200 mL^63^. The organisms were counted according to Uthermőhl^58^, by observing the entire area of the sedimentation chamber with an inverted microscope (Axiovert 35, Zeiss) at 100× and 400×. One replicate was processed for each sample. Organisms were measured and grouped according to size and standardized geometrical forms. The biovolume of non-loricate ciliates was calculated according to Edler^64^; the biovolume of nauplii and copepods were calculated according to Ruttner-Kolisko^65^ and Shmeleva^66^, while for tintinnids biovolume was estimated by measuring the linear dimensions of their lorica. The biovolume of nauplii and copepods were calculated according to Ruttner-Kolisko^65^ and Shmeleva^66^ respectively. The following conversion factors were used to transform the biovolume into carbon biomass: for non-loricate ciliates 0.14 pg C μm^−3 ^^67^, for tintinnids 444.5 pg C μm^−3^ (lorica volume in μm^−3^ × 0.053 pg C) according to Verity and Langdon^68^, for copepod nauplii and post-naupliar copepods 0.08 pg C μm^−3 ^^69,70^.

### Statistical analyses

Differences among water masses in term of biomass of different heterotrophic and autotrophic plankton cells size classes and abundance of phytoplankton species were tested by the analysis of similarities (ANOSIM)^71^. Before the analysis, all plankton biomass data were double square transformed. Since in both cases ANOSIM revealed significant differences among water masses, matrices were then used to produce Non-metric MultiDimensional Scaling (NMDS) plots. The coefficients of dissimilarity and phytoplankton species responsible for the clustering were determined by means of SIMPER analyses. ANOSIM, NMDS plot and SIMPER analyses were carried out with PRIMER v5.2.2 (Plymouth Marine Laboratory, UK). Linear correlation analyses were performed using Statistica by Statsoft (Kernel release 5.5).

### Hydrodynamic and Lagrangian modelling

The simulations of the 2012 CAO event were performed by coupling the *Regional Ocean Modelling System* (ROMS^72^, www.myroms.org) for hydrodynamics and the *Simulating Waves Nearshore* model (SWAN)^73^ for waves, through a *Coupled–Ocean–Atmosphere–Wave–Sediment–Transport system* (COAWST)^74^. The modelling domain covers the whole Adriatic Sea with an open boundary at the Otranto Strait, a 1 km horizontal grid and 30 stretched sigma vertical levels (maximum resolution near surface and bottom). The simulation was initialized on November 1^st^ 2011 and lasted until June 28^th^ 2012 to cover the CAO event and its preconditioning phase. Models were forced at surface with atmospherical fields derived from a COSMO implementation run by the Emilia Romagna Environmental Protection Agency (COSMO-I7)^75^. COSMO-I7 is a non-hydrostatic, 3-D numerical weather prediction model with a 7.0 × 7.0 km^2^ horizontal resolution and 35 vertical terrain-following levels. A detailed description of set up and validation can be found in Benettazzo *et al*.^22^ and Carniel *et al*.^76^.

The Lagrangian dispersion of *Skeletonema* sp. was studied with an individual based Lagrangian tracking model (ICHTHYOP)^77^ in which virtual diatoms (VDs) had shape, dimension and buoyancy similar to a single cell of *Skeletonema* sp. (prolapsed ellipsoids having average major and minor axis of 6.2 and 4 µm respectively, and density of 1070 kg m^−3^)^78^. A total of 200,000 VDs were released in the northern Adriatic on February 5^th^ during a large *Skeletonema* bloom, as shown by satellite images (Supplementary Fig. S3) and modelling results (data not shown), and in the middle of CAO, hence giving the opportunity to follow a maximum dispersion event. Particles were released over the whole dense water generation site, located roughly north of Rimini (Fig. 1), at depths shallower than the 30 m isobaths and left to drift according to buoyancy and current fields derived from the COAWST simulations. The numerical simulation lasted for 100 days.

To identify the pathways followed by the VDs the modelling outputs were decomposed on a 3D reference grid (2 km × 2 km horizontal cells, and 61 vertical layers each 20 m thick) and merged to obtain a distribution of the probability of passage. Analyses were carried out on 4 transects: Rimini (TR), Ancona (TA) and San Benedetto del Tronto (TB, according to Benetazzo *et al*.)^22^ and Gargano (TG) positioned directly over station 29 in front of the Gargano peninsula (in Benetazzo *et al*.^22^ this transect was located ca. 30 km north of station 29). The total number of particles crossing each transect per day was computed and their distribution used to identify the dense water mass that directly carried the VDs. The limits of the VDs distribution area were defined with a Delaunay triangulation. Fluxes for water masses denser than 1029.3 kg m^−3^ were then computed daily (every 6 hours) for both the whole transect area (Total flux, TF) and for the area specifically identified by the presence of VDs (Reduced Flux, RF). A detailed description of the Lagrangian model simulation and analysis is available in supplementary material S4.

### Data availability

The datasets generated during and/or analysed during the current study are available from the corresponding author on reasonable request.

## Electronic supplementary material

Supplementary information
