# Supplementary material for: Massive shelf dense water flow influences plankton community structure and particle transport over long distance
Source: Sci Rep. 2018 Mar 14;8:4554. doi: 10.1038/s41598-018-22569-2 (PMC5852251; doi:10.1038/s41598-018-22569-2)
Supplement: Supplementary file 1 — Supplementary information [file 41598_2018_22569_MOESM1_ESM.docx]

**Massive shelf dense water flow influences plankton community structure and particle transport over long distance**

Fabrizio Bernardi Aubry^a^, Francesco Marcello Falcieri^a^, Jacopo Chiggiato^a^, Alfredo Boldrin^a^, Gian Marco Luna^b^, Stefania Finotto^a^, Elisa Camatti^a^, Francesco Acri^a^, Mauro Sclavo^a^, Sandro Carniel^a^, Lucia Bongiorni^a *^

^a^Institute of Marine Sciences, National Research Council, Arsenale - Tesa 104, Castello 2737/F

30122, Venice, Italy

^b^Institute of Marine Sciences, National Research Council, Largo Fiera della Pesca 2, 60125 Ancona, Italy

**Supplementary information**

**Table S1.**Station number, longitude, latitude, bottom depth and corresponding water mass typology: WACC (Western Adriatic Coastal Current), OUL (Offshore Upper Layer), MLIW (Modified Levantine Intermediate Water), lNAdDW (Northern Adriatic Dense Water, low content), mNAdDW (Northern Adriatic Dense Water, medium content), hNAdDW (Northern Adriatic Dense Water, high content), nADW (newly generated Adriatic Deep Water) and oADW (old, resident Adriatic Deep Water).

| **Station number** | **Longitude [E]** | **Latitude [N]** | **Depth [m]** | **Water mass** |
| --- | --- | --- | --- | --- |
| 29 | 16.668 | 41.936 | 0.5  141 | OUL (surface)  hNAdDW (bottom) |
| 42 | 16.455 | 42.065 | 0.5  25  128 | OUL (surface)  Shelf (DCM)  hNAdDW (bottom) |
| 44 | 16.356 | 41.959 | 0.5  15  78 | WACC (surface)  WACC (DCM)  lNAdDW (bottom) |
| 46 | 16.278 | 41.869 | 0.5 | WACC (surface) |
| 43b | 16.414 | 42.024 | 0.5  25  113 | OUL (surface)  Shelf (DCM)  hNAdDW (bottom) |
| 60 | 16.476 | 41.758 | 0.5  82 | WACC (surface)  lNAdDW (bottom) |
| 68 | 16.886 | 41.886 | 0.5  367 | OUL (surface)  nADW (bottom) |
| 71 | 17.031 | 41.931 | 0.5  600 | OUL (surface)  nADW (bottom) |
| 91 | 17.692 | 41.743 | 0.5  26  1194 | OUL (surface)  OUL (DCM)  oADW (bottom) |
| 93 | 17.338 | 41.563 | 0.5  25 | OUL (surface)  OUL (DCM) |
| 94 | 17.161 | 41.451 | 0.5  48 | OUL (surface)  OUL (DCM) |
| 14_2 | 17.078 | 41.394 | 0.5 | OUL (surface) |
|  |  |  | 173 | MLIW (bottom) |
| 1_2b | 17.004 | 41.346 | 133 | MLIW (bottom) |

**Table S2.** Mean values of the main hydrological variables, nutrient concentrations and value of biomass of different heterotrophic and autotrophic plankton size classes in the analysed surface/subsurface and deep water masses. Total Suspended Matter (TSM); Autotrophic (A_); Heterotrophic (H_); Heterotrophic to Autotrophic biomass ratio (H/A).

|  | **SURFACE - SUBSURFACE WATERS** | | | **DEEP WATERS** | | | | |
| --- | --- | --- | --- | --- | --- | --- | --- | --- |
|  | **OUL** | **Shelf** | **WACC** | **hNAdDW** | **lNAdDW** | **MLIW** | **nADW** | **oADW** |
| **Temperature (°C)** | 14.67 | 13.96 | 13.48 | 11.02 | 12.55 | 13.51 | 13.2 | 13.24 |
| **Salinity** | 38.77 | 38.76 | 38.43 | 38.64 | 38.68 | 38.71 | 38.7 | 38.73 |
| **Turbidity (FTU)** | 40.2 | 24.81 | 41.96 | 93.43 | 147.14 | 47.52 | 66.96 | 10.59 |
| **Oxygen (%)** | 104.63 | 97.99 | 102.09 | 93.9 | 96.93 | 90.33 | 92.12 | 79.98 |
| **Density anomaly (Kg m^-3^)** | 28.95 | 29.1 | 28.95 | 29.6 | 29.34 | 29.16 | 29.22 | 29.23 |
| **TSM (mg L^-1^)** | 0.79 | 0.83 | 3.66 | 3.18 | 2.24 | 0.57 | 0.69 | 0.25 |
| **Chlorophyll-a (µg L^-1^)** | 0.93 | 1.64 | 0.46 | 0.19 | 0.41 | 0.12 | 0.27 | 0.22 |
| **DIN (µM)** | 0.79 | 1.59 | 1.65 | 2.35 | 1.39 | 3.39 | 2.85 | 4.54 |
| **Si-SIO_4_ (µM)** | 0.18 | 1.17 | 0.62 | 2.65 | 1.7 | 2.48 | 2.48 | 8.87 |
| **P-PO_4_ (µM)** | 0.02 | 0.01 | 0.01 | 0.03 | 0.03 | 0.09 | 0.05 | 0.17 |
| **A_ pico (µgC L^-1^)** | 0.56 | 0.16 | 0.83 | 0.25 | 0.44 | 1.23 | 0.13 | 0 |
| **A_ nano (µgC L^-1^)** | 12.22 | 10.49 | 6.6 | 1.14 | 1.28 | 0.95 | 1.04 | 0.24 |
| **A_ micro (µgC L^-1^)** | 70.54 | 70.01 | 51.02 | 6.07 | 13.46 | 6.79 | 4.59 | 0.02 |
| **H_ pico (µgC L^-1^)** | 2.9 | 1.99 | 1.64 | 1.66 | 1.78 | 0.57 | 0.98 | 0.38 |
| **H_ nano (µgC L^-1^)** | 0.51 | 0.36 | 1.01 | 0.61 | 0.51 | 0.22 | 0.48 | 0.23 |
| **H_ micro (µgC L^-1^)** | 1.29 | 0.38 | 1.51 | 0.32 | 0.84 | 0.05 | 0.04 | 0.1 |
| **H/A ratio** | 0.06 | 0.05 | 0.09 | 0.46 | 0.21 | 0.08 | 0.34 | 2.65 |

**
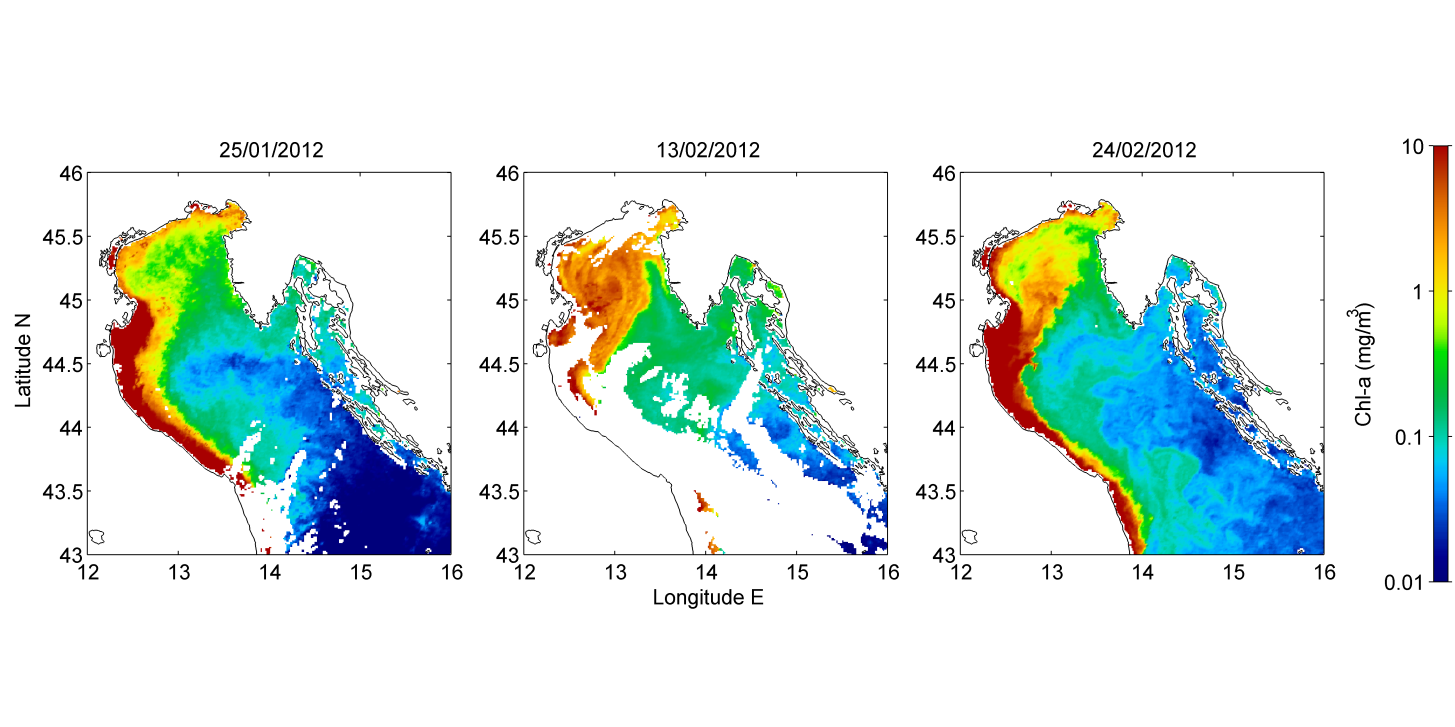
Figure S3.** Daily multichannel L3 ocean color chlorophyll for 25 January, 13 February, 24 February 2012, respectively before, during and after the CAO. Data courtesy of Copernicus Marine environment monitoring service (<http://marine.copernicus.eu/>*),* maps generated using MATLAB 7.1 (<http://uk.mathworks.com/products/matlab>).

**Supplementary material S3**

The dispersion due to currents and buoyancy of *Skeletonema* sp. from its blooming site was investigated with a set of Lagrangian numerical simulation. The model used, ICHTHYOP (Lett et al., 2008), was first developed to study the dispersion and development of fish eggs and larvae in marine environments. In this work we do not focus on diatoms growth or on biological cycles but just on their dispersion, hence the model was implemented with all its biological features (i.e. larval growth, recruitment of juveniles, diurnal vertical migrations, lethal temperature and mortality) deactivated. This way the VDs disperse as a purely Lagrangian drifter (i.e. under the effect of horizontal/vertical advection and dispersion) but are also influenced by a buoyancy force resulting from the difference between particle and environment densities added to the vertical current velocities. For each time step the individuals displacement of particles was computed with a Runge-Kutta 4 integration scheme; horizontal dispersion was included (for details see Peliz et al., 2007) with a turbulent dissipation rate of є=10^-9^; a value in agreement with turbulent kinetic energy observations in the Adriatic Sea (Falcieri et al., 2016, Carniel et al., 2012).

In the results presented here, the Lagrangian simulations were done with diatoms colonies formed by single cell with the shape of a prolapsed ellipsoid (major axis of 6.2 µm and a minor axis of 4 µm). Instead, the samples collected at station 29 most of S*keletonema* sp. were in colonies of 1 to 5 cells.

In order to cope with possible differences due to colonies length, test cases were implemented with chains length ranging from 1 to 5 cells. The colonies diameter was kept constant in all simulation while the length was set so to consider both the number of cells and the gaps (which are filled by processes with a connection role) between one cell and the other. Each simulation was initialized on February 14^th^ with the instantaneous release of 30000 particles over the same area used for the simulations of February 5^th^ at a depth between 5 and 35 m. Particles were tracked for 100 days. Results (Fig. S4) show that there is no significant difference in the timing and number of passages across transects or in the distribution of pathways. The same approach was used to test sensitivity to cell weight by changing the density of the VDs of each length class across a spectrum of densities ranging from 1029.5 kg m^-3^ to 1070 kg m^-3^; again no significant differences were found among simulations. For the main simulation with release set on February 5^th^diatoms were represented by a single cell with density of 1070kg m^-3^. VDs were released over and area covering the whole northern Adriatic (southern limit corresponding to the -30m isobath) which satellite images shows as the most prone to phytoplankton blooming events.

First step of post-processing was to identify the VDs that crossed each reference transect and to decompose their trajectories over a 3D reference grid (1km x 1km with 20m thick vertical layers). Then VDs passing through each transect in sites with density higher than 1029.3 kg m^-3^ were identified and used to computed the pathways distribution maps. Two types of water transport were computed for each transect: one considering all the water denser than 1029.3 kg m^-3^, representative of the overall export, and a reduced one with just the VDs carrying waters. Those waters were defined as the dense waters inside and area identified with a Delauny triangulation computed over all the daily passages across a given transect.

**Figure S4.** Time of VDs passage across TG in the test-cases implemented before simulations. Each color represents a VD colony length class from 1 cell (R1) to 5 cell (R5).


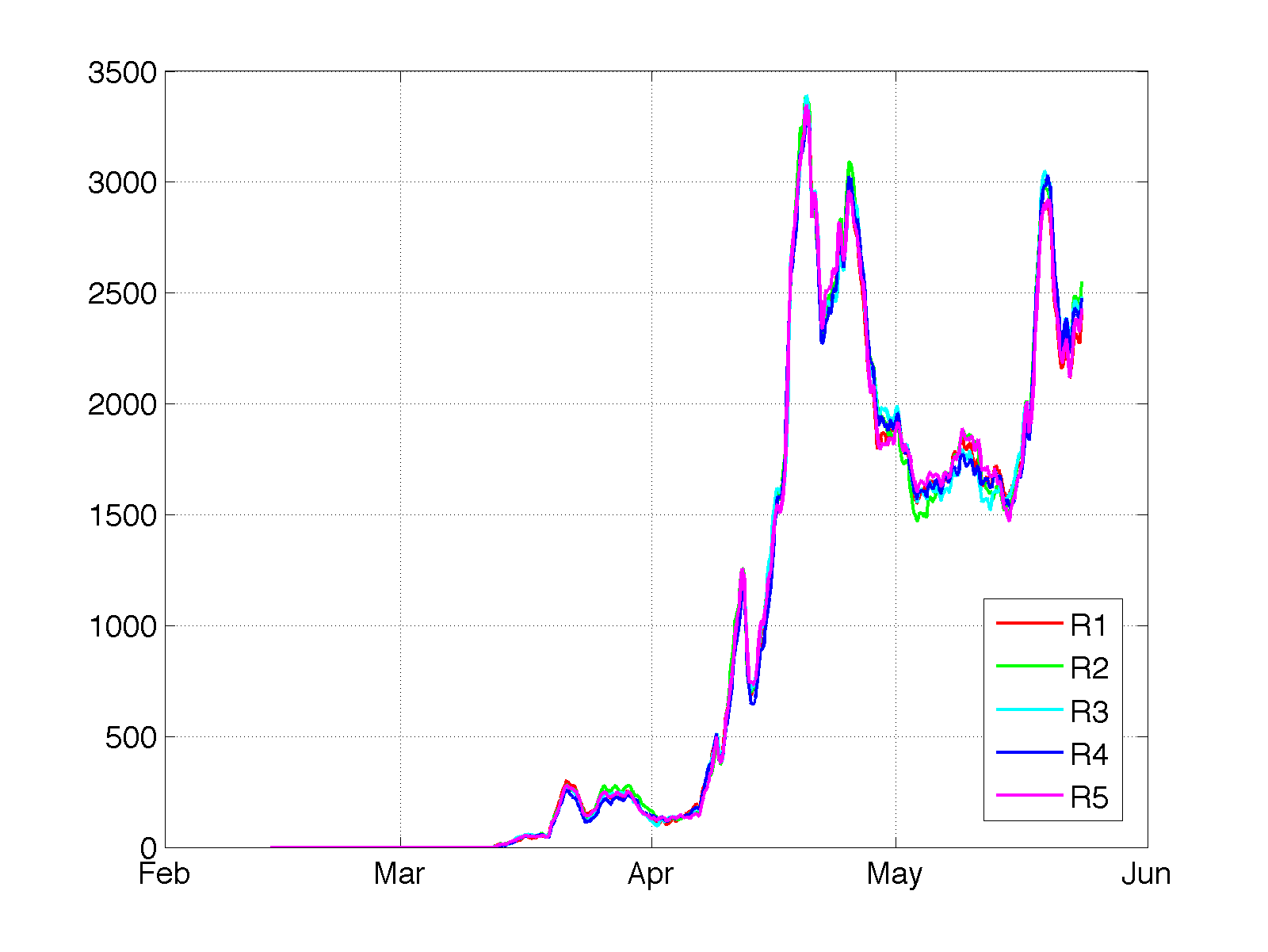


**
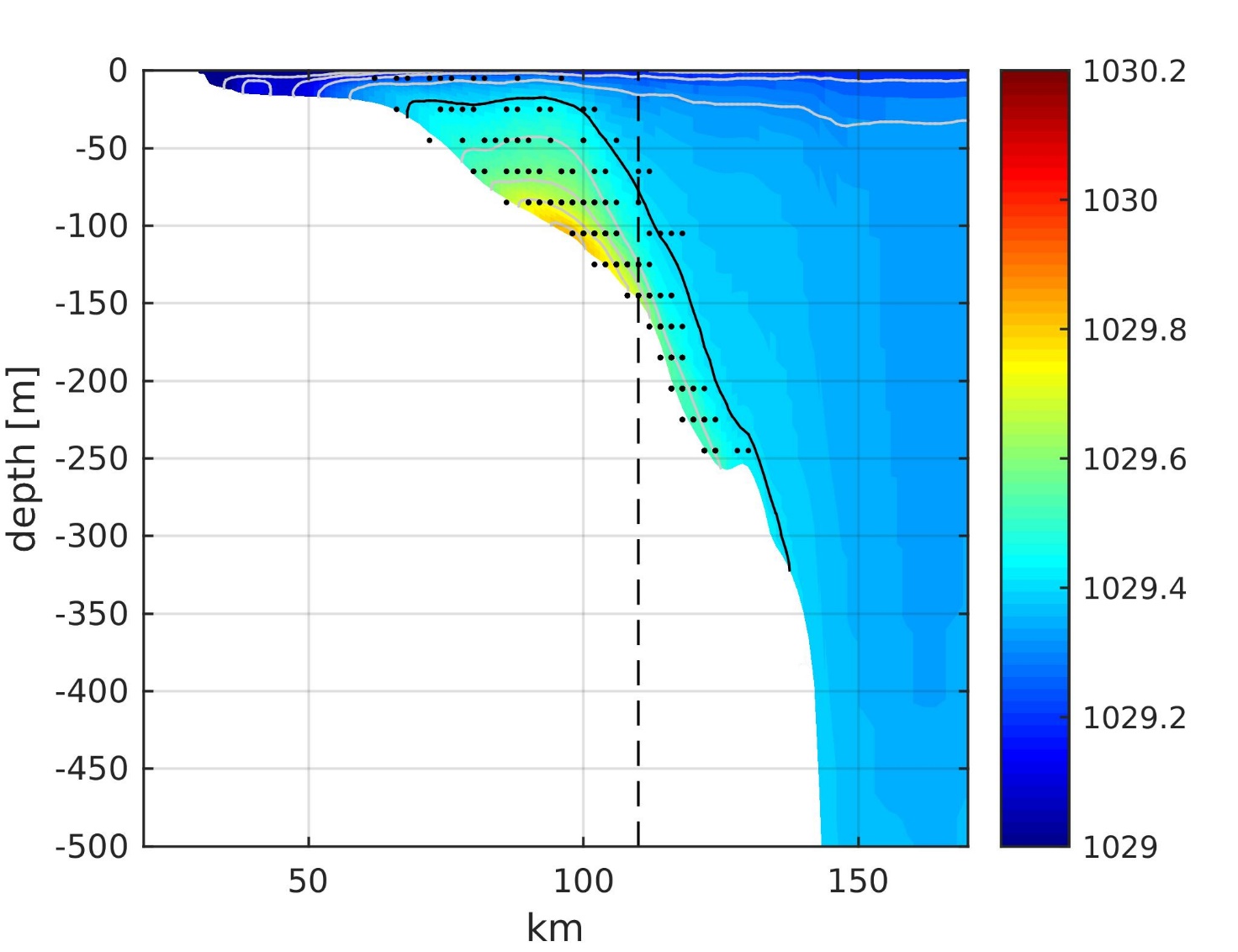
**

**Figure S5.** Average density distribution at the Gargano transect during the 5 days interval centered on March 24^th^. The black contour line shows the 1029.4 Kg m^-3^ density limit, the vertical dashed line the position of station 29. Black dots mark the position of transit of VDs. The figure shows how the model not only correctly represents a higher number of passages in the proximity of station 29 but also does not show significant passages outside the core of the DW vein, in accordance to low abundance of *Skeletonema* found in non-dense waters stations.
